# Physical Activity Behaviors and Barriers in Multifetal Pregnancy: What to Expect When You’re Expecting More

**DOI:** 10.3390/ijerph18083907

**Published:** 2021-04-08

**Authors:** Victoria L. Meah, Morgan C. Strynadka, Rshmi Khurana, Margie H. Davenport

**Affiliations:** 1 Program for Pregnancy and Postpartum Health, Physical Activity and Diabetes Laboratory, Faculty of Kinesiology, Sport, and Recreation, Women and Children’s Health Research Institute, Alberta Diabetes Institute, University of Alberta, Edmonton, AB T6G 2E1, Canada; vmeah@ualberta.ca (V.L.M.); mstrynad@ualberta.ca (M.C.S.); 2 Departments of Medicine and Obstetrics & Gynecology, Women and Children’s Health Research Institute, University of Alberta, Edmonton, AB T6G 2G3, Canada; Rshmi.Khurana@albertahealthservices.ca

**Keywords:** exercise, multiple pregnancy, activity restriction, healthcare provider advice

## Abstract

The health benefits of prenatal physical activity (PA) are established for singleton pregnancies. In contrast, individuals with multifetal pregnancies (twins, triplets or more) are recommended to restrict or cease PA. The objectives of the current study were to determine behaviors and barriers to PA in multifetal pregnancies. Between 29 May and 24 July 2020, individuals with multifetal pregnancies participated in an online survey. Of the 415 respondents, there were 366 (88%) twin, 45 (11%) triplet and 4 (1%) quadruplet pregnancies. Twenty-seven percent (*n* = 104/388) of respondents completed no PA at all during pregnancy, 57% (*n* = 220/388) completed PA below current recommendations, and 16% (*n* = 64/388) achieved current recommendations (150-min per week of moderate-intensity activity). Most respondents (*n* = 314/363 [87%]) perceived barriers to PA during multifetal pregnancy. The most prominent were physical symptoms (*n* = 204/363 [56%]) and concerns about risks to fetal wellbeing (*n* = 128/363 [35%]). Sixty percent (*n* = 92/153) felt that these barriers could be overcome but expressed the need for evidence-based information regarding PA in multifetal pregnancy. Individuals with multifetal pregnancies have low engagement with current PA recommendations but remain physically active in some capacity. There are physical and psychosocial barriers to PA in multifetal pregnancy and future research should focus on how these can be removed.

## 1. Introduction

Multifetal births, including twins, triplets, and high-order pregnancies, account for approximately 3–4% of all births [1]. In multifetal pregnancies, the risk of developing pregnancy complications, such as hypertensive disorders of pregnancy, gestational diabetes, and preterm delivery, rises in direct proportion to the number of fetuses [2]. Recent guidelines demonstrate the powerful influence of physical activity in preventing or treating some of these complications [3]. Pregnant individuals achieving 150-min per week of moderate-intensity exercise had reduced odds of preeclampsia (by 41%), gestational hypertension (by 39%), gestational diabetes mellitus (by 38%), excessive gestational weight gain (by 32%), and prenatal depression (by 67%); with no increased risk of adverse outcomes such as preterm labor or fetal mortality [4,5,6,7,8]. However, these health benefits are only established for those with singleton pregnancies.

Physical activity describes a spectrum of movement behaviors including moderate-to-vigorous intensity activity (e.g., exercise), light activities (e.g., gentle walking and daily activities) and sedentary behaviors (e.g., sitting/screen-time). Considering the established health benefits of prenatal exercise, those with uncomplicated singleton pregnancies are encouraged to complete 150-min of moderate intensity physical activity per week [9,10,11]. In contrast, guidelines recommend individuals with high-order multifetal pregnancies avoid moderate-to-vigorous intensity physical activity throughout gestation (absolute contraindication) and twin pregnancies are advised to speak to their healthcare provider about remaining active after 28 weeks gestation (relative contraindication) [9,10,11]. However, our recent systematic review underscores the lack of empirical research supporting the inclusion of multifetal pregnancies as a contraindication to physical activity [12]. Further, the prophylactic prescription of activity restriction is a common recommendation for this population to reduce risk despite compelling evidence that activity restriction, and/or bed rest, does not improve maternal or fetal outcomes [13,14,15,16].

Barriers to physical activity are factors that prevent an individual from participating in movement or exercise. In the general population, barriers to physical activity may include lack of time, lack of access, and/or psychological factors such as poor motivation. Barriers to physical activity in multifetal pregnancy have not been investigated. However, individuals with multifetal pregnancies have greater anatomical (e.g., large extended abdomen [17], musculoskeletal pain) and physiological (e.g., cardiorespiratory demands, fatigue) constraints as well as psychological factors (e.g., increased stress [18] and perceived risk of complications) that may influence physical activity behavior beyond the typical barriers experienced by singleton pregnancies [19]. Additionally, healthcare providers, who play a significant role in supporting positive lifestyle behaviors during pregnancy [20,21], often provide physical activity guidance to those with multifetal pregnancies that is focused on activity restriction, not promotion [22]. Therefore, individuals with multifetal pregnancies may face various barriers that prevent them from deriving the health benefits of physical activity. At present, little is known about the actual experiences of and barriers to prenatal physical activity in multifetal pregnancy.

The objectives of the current study were to: (1) determine physical activity behaviors in individuals with multifetal pregnancies; (2) examine barriers to physical activity in multifetal pregnancy; and (3) generate participant-led suggestions for physical activity research in multifetal pregnancies.

## 2. Materials and Methods

### 2.1. Ethical Approval and Participant Recruitment

This study was approved by the University of Alberta Health Research Ethics Board (Pro00093321) and was conducted in accordance with the Declaration of Helsinki, apart from registration in a publicly available database. Between 29 May to 24 July 2020, participants who were currently or previously pregnant with a multifetal pregnancy were invited to participate in a questionnaire. The questionnaire was available through an online platform, thereby allowing easy access for individuals with multifetal pregnancies to participate in the study. Recruitment was achieved via a mixture of convenience and snowball sampling. This was achieved through sharing the questionnaire within the authors’ professional networks, on social media platforms, and direct contact to associations for parents with multifetal births. All participants provided electronic informed consent; however, all responses to the questionnaire were anonymous. Data were collected using Research Electronic Data Capture, a secure, web-based software designed to support data capture for research studies, hosted by the Women and Children’s Health Research Institute, University of Alberta, Canada.

### 2.2. Questionnaire Content

The questionnaire was developed and revised by researchers (M.H.D., V.L.M., M.C.S.), a medical professional and patient advocate (R.K.). A copy of the complete questionnaire is included in the Supplement. Participants were informed of the purpose of the survey and provided voluntary electronic informed consent. Eligibility was confirmed through questions regarding multifetal gestation (e.g., are you having or have you had a multifetal pregnancy). Participants who met the inclusion criteria were invited to answer questions on maternal demographics (e.g., age, anthropometry, ethnicity, sociodemographic factors, pre-pregnancy physical activity); birth and infant outcomes; prenatal physical activity behavior; knowledge and sources of information, barriers to activity; activity restriction prescription and experience; as well as interest and willingness to participate in research. For the latter, a 5-point Likert scale (ranging from “Not at all” to “A lot”) was used to assess factors that may influence the likelihood of participation in future prenatal physical activity research. All sections included multiple choice questions but concluded with open-ended questions allowing participants to share further information if they wished to do so.

Physical activity was self-reported in two ways. First, participants provided an overall assessment of their achievement of 150-min of moderate-intensity physical activity each week (i.e., current Canadian recommendations for pregnant and postpartum women) [9,10,11]. Secondly, participants reported details of frequency (1 to 7 days per week), intensity (light, moderate, or strenuous) and duration of physical activity regularly completed during the week. Activities were assigned a metabolic equivalency (MET) score using the Compendium of Physical Activity [23]; and were multiplied by frequency and duration to determine volume of exercise (MET·minutes·week^−1^), as previously described [24].

Activity restriction was defined in this study as any reduction or cessation of physical activity as recommended by a healthcare professional. This included modification of physical activity (reduction in frequency, intensity, type, and time), avoidance of lower body exercise (known as pelvic rest), avoidance of physical activity but maintenance of activities of daily living, and finally, avoidance of all activity, more commonly known as bed rest.

### 2.3. Questionnaire Analysis

Following closure of the questionnaire, all data were checked for accuracy and invalid data were removed. Invalid data was considered as empty or incomplete answers or impossible answers (e.g., nonhuman weight). All exclusions were independently reviewed by two researchers (V.L.M. and M.C.S.) and discarded following consensus.

#### 2.3.1. Quantitative Data Analysis

The frequency of responses (*n* and % of total cohort) and mean ± standard deviation (SD), where appropriate, were calculated for numerically coded questions. Statistical comparisons (α = 0.05) were made as follows. One-way ANOVA and post hoc Bonferroni comparisons were made for intensity of activity and gestational age at activity cessation. Additionally, participants were separated into subgroups for additional analyses: (1) twin vs. high-order pregnancies and (2) complicated vs. uncomplicated pregnancies. “Uncomplicated” refers to participants that reported no pre-existing or prenatal medical conditions that would contraindicate physical activity; “complicated” refers to pre-existing conditions or development of pregnancy-specific maternal or fetal complications during multifetal pregnancy (e.g., hypertensive disorders, gestational diabetes, preterm labor, growth restriction, etc.) that would contraindicate physical activity in this population. To determine differences between groups, tests of two proportions were used for frequency data and multiple t-tests using the false discovery rate were used for comparisons of physical activity. Assumptions of statistical tests were checked and met.

#### 2.3.2. Qualitative Data Analysis

Open-ended questions were analyzed using theoretical thematic analysis [25]. A contextualist approach was used to determine themes within the data, respecting that certain aspects of pregnancy and its physiology may contribute as a barrier to physical activity, but also that other aspects of society may influence the experiences of each participant [25]. Themes of the questionnaire: Economic, Environmental or Access, Psychological, Social, Healthcare Provider, Physical, Comorbid Conditions, Information, and Prior Experience were generated a priori. An additional category “Other” was also included for factors that arose that did not fall within the a priori themes—only one additional theme was identified—“Work”. Additionally, impressions (positive, negative, or combined) of activity restriction were identified. Two researchers (V.L.M. and M.C.S.) independently coded all qualitative answers and data were compared to confirm consensus. Disagreements were addressed through discussion. Prominent themes were identified through the frequency of responses (*n* and % of total cohort) and representative quotes were identified to support the broad topics discussed by respondents.

## 3. Results

**Figure 1:**
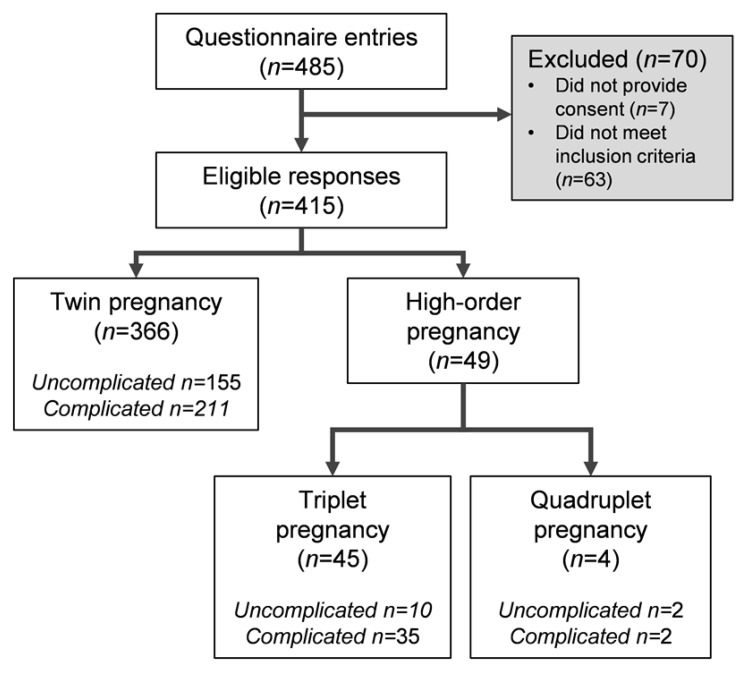
Study recruitment flowchart. Responses were excluded if: full consent was not provided, participants reported having only a singleton pregnancy or incomplete data with no responses to physical activity questions were submitted. “Uncomplicated” refers to participants that reported no pre-existing or prenatal medical conditions that would contraindicate physical activity; “complicated” refers to pre-existing conditions or development of pregnancy-specific maternal or fetal complications during multifetal pregnancy (e.g., hypertensive disorders, gestational diabetes, preterm labor, growth restriction, etc.) that would contraindicate physical activity in this population.

In total, 415 respondents provided consent and met inclusion criteria (Figure 1). As all questions were voluntary, response rates differed between questions, and as such, the total number of participants included in each analysis are provided within the following text or tables.

### 3.1. Maternal Demographics

Participant demographics are provided in Table 1. Of the 415 respondents, 8% had current (*n* = 33; gestational age at assessment: 26.3 ± 5.8 weeks) and 93% had previous multifetal pregnancies (*n* = 382; postdelivery: 4.7 ± 6.7 years). Most participants had no medical conditions prior to pregnancy (*n* = 228/379 [60%]) or conditions that would have not impacted exercise prescription (e.g., anxiety [15%], depression [8%] or categorization as overweight/obese [9%]). Fourteen percent of respondents (*n* = 54/379) had pre-existing complications that may have been considered a contraindication to prenatal exercise in conjunction with multifetal pregnancy (e.g., type 1 diabetes mellitus [1%], respiratory [3%], thyroid [3%], cardiovascular [2%] or musculoskeletal [1%] disorders). Most respondents conceived spontaneously (*n* = 269/413 [65%] vs. with assistance from reproductive therapies [35%]). During pregnancy, 59% of respondents developed a maternal complication (*n* = 236/401; Table 1).

### 3.2. Birth and Infant Outcomes

Most respondents with prior multifetal pregnancies gave birth via a planned delivery (*n* = 185/381 [49%]) with lower rates of spontaneous [25%] and emergency [27%] deliveries. Thirty-eight percent of infants were not diagnosed with any complications (*n* = 140/371). However, of those reporting one or more neonatal complications there were cases of neonatal jaundice (35%), respiratory problems (26%), small-for-gestational age (24%), growth restriction (9%), hypoglycemia (9%), infection (5%), twin-to-twin transfusion syndrome (5%) and/or congenital defects (5%). Over half of respondents reported that one or more of infants were admitted to the neonatal intensive care unit at birth (*n* = 200/382; 52%).

### 3.3. Prenatal Physical Activity Knowledge

Most respondents believed individuals with multifetal pregnancies should be active during their pregnancies (*n* = 380/414 [92%]; Table S1). Respondents thought that prenatal activity should be completed at light (*n* = 270/412 [66%]) and moderate-to-vigorous intensities (*n* = 160/412 [39%]), on three or more days per week (*n* = 328/414 [79%]). The majority of respondents thought they should participate in physical activity for less than 150-min per week (*n* = 198/404 [49%]), 45% (*n* = 180) thought activity should be done for 150-min or more during pregnancy, but 6% (*n* = 26) thought they should not be active at all. The most common activities that were perceived as “okay” to complete during multifetal pregnancy were walking (*n* = 402/413 [97%]), swimming (*n* = 307 [74%]), stretching (*n* = 305 [74%]), and yoga (*n* = 286 [69%]).

Information regarding physical activity during multifetal pregnancy most commonly came from healthcare providers (*n* = 258/409 [62%]). Respondents reported specific discussions regarding physical activity with their obstetrician (*n* = 206/258 [79%]), midwife (*n* = 54 [21%]), or family doctor (*n* = 50 [19%]), although other healthcare professionals, including nurses, physiotherapists, maternal-fetal medicine specialists, and dieticians, also provided information. Recommendations for physical activity provided by healthcare providers were mostly nonspecific (*n* = 175/237 [74%], e.g., “listen to body”, “do what feels comfortable” or “do not overdo it”) but some information was in line with current singleton physical activity guidelines (*n* = 52/237 [22%], e.g., “moderate exercise okay, as long as I could carry on a conversation” and “avoid contact sports and activities with risk of falls”.

Respondents also used other sources to gain information on prenatal physical activity (*n* = 258/392 [66%]). Books (30%), social media (24%), healthcare websites (21%), family/friends (17%), and pregnancy support groups (17%) were utilized; however, much of this information was not specific to multifetal pregnancy. Only 31% of respondents looking at these nonhealth care provider sources (*n* = 66/210) found information that was directly relevant to physical activity in their twin or high-order pregnancy. When asked what information they wished they had known about physical activity during their multifetal pregnancy, respondents gave answers such as “Evidence-based guidelines for high-order multiples, not just singletons or twins”, “Everything! This topic is not addressed well AT all!” and “That it was safe. I did not have to be so scared to be active”.

### 3.4. Prenatal Physical Activity Bevahiours

Prior to pregnancy, 43% of respondents reported being physically active in accordance with current guidelines most, if not all, of the time (Table 2). In contrast, only 16% reported meeting guidelines for physical activity during pregnancy, with 27% indicating no physical activity at all during their multifetal pregnancy (Table 2). Average self-reported frequency, intensity, duration, and volume of activity for all respondents are shown in Table 2.

Light and moderate intensity activities were more frequently reported, with cessation of strenuous (22 ± 10 weeks, *p* < 0.001) and moderate intensity physical activity (29 ± 9 weeks, *p* = 0.012) earlier in pregnancy compared with light activities (31 ± 8 weeks). Most activities were completed in a recreational facility, although many participants also reported being active at home or outdoors in their neighborhood.

#### Subgroup Analyses

Twin and high-order pregnancies had similar levels of prepregnancy physical activity, although a higher proportion of high-order pregnancies reported no prenatal activity at all (*p* = 0.002; Table S2). There were no significant differences in self-reported intensity (adjusted *p* = 0.43), frequency (adjusted *p* = 0.17), duration (adjusted *p* = 0.17), volume (adjusted *p* = 0.73) or gestational age at activity cessation (all intensities; adjusted *p* = 0.17) between high-order and twin pregnancies (Table S2).

Uncomplicated and complicated pregnancies had similar levels of prepregnancy physical activity, although a higher proportion of complicated pregnancies reported no prenatal activity at all (uncomplicated: *n* = 29/154 [19%] vs. complicated *n* = 75/234 [32%]; *p* < 0.001; Table S3). Of those that did report being physically active, a significantly lower proportion of individuals with complicated pregnancies achieved 500 or more MET·mins·week^−1^ compared to those with uncomplicated multifetal pregnancies (*n* = 113/175 [65%] vs. *n* = 96/124 [77%], respectively; *p* = 0.017). Additionally, average duration (38 ± 20 vs. 45 ± 30 min; adjusted *p* = 0.013) and volume of activity (1073 ± 987 vs. 1625 ± 1957 MET·mins·week^−1^; adjusted *p* = 0.003) were significantly lower in complicated vs. uncomplicated pregnancies, but there were no differences between groups in self-reported intensity (adjusted *p* = 0.11) or frequency (adjusted *p* = 0.24). Gestational age at activity cessation (all intensities) was significantly earlier in complicated vs. uncomplicated pregnancies (29 ± 8 and 31 ± 8 weeks, respectively; adjusted *p* = 0.016; Table S3).

### 3.5. Barriers to Physical Activity

Most respondents (*n* = 314/363 [87%]) perceived barriers to physical activity during their multifetal pregnancy. The most prominent barrier to remaining physically active was the presence of physical symptoms. When specifically asked what physical symptoms limited respondent’s ability to be physically active, various factors ranging from fatigue, pain, nausea/vomiting, shortness of breath, and stress urinary incontinence were reported (Table 3). Additionally, nearly two thirds of respondents (*n* = 239/369 [65%]) reduced their physical activity by choice, without formal prescription from a healthcare provider. Of this group, most respondents reported that they made this decision due to physical symptoms (*n* = 179/211 [85%]), and some reported concerns about fetal wellbeing (*n* = 46/211 [22%]).

When asked if there was anything that would have helped participants overcome barriers to physical activity, 40% (*n* = 61/153) believed nothing would have helped. However, 60% (*n* = 92) of respondents did provide suggestions for how to remove barriers for physical activity in multifetal pregnancy (Table 3).

#### Subgroup Analyses

The barriers to physical activity were relatively similar between twin and high-order pregnancies (Table S4). However, individuals with high-order pregnancies reported healthcare provider advice against physical activity (*n* = 19/42 [45%] vs. *n* = 64/321 [20%]; *p* < 0.001) and exhaustion (*n* = 27/42 [64%] vs. *n* = 192/341 [56%]; *p* = 0.006) more frequently as a barrier when compared with twin pregnancies.

### 3.6. Activity Restriction and Experience

During multifetal pregnancy, 44% of respondents (*n* = 169/380) were prescribed activity restriction by their healthcare provider (modification or cessation of activity, or bed rest); however, only 38% (*n* = 144/380) knew why they were given this prescription. When considering the different categories of activity restriction, 16% of multifetal pregnancies were prescribed bed rest (*n* = 62/380; average: 25.2 ± 7.0 weeks), 15% were prescribed avoidance of physical activity (*n* = 58; average 23.4 ± 7.2 weeks), 14% were prescribed pelvic rest (*n* = 55; average 19.4 ± 7.6 weeks) and 24% were prescribed physical activity modification (reduction in frequency, intensity, type, and time of physical activity; *n* = 90, average 22.8 ± 8.3 weeks). When asked about prescription of any activity restriction, most respondents described the experience negatively; however, this was varied (Table 4; Table S5).

#### Subgroup Analyses

Prescription of any type of activity restriction was more frequent in high-order pregnancies compared with twin pregnancies (*n* = 27/43 [63%] vs. *n* = 142/337 [42%], *p* = 0.010). Specifically, a larger proportion of high-order pregnancies were prescribed bed rest compared with twin pregnancies (*n* = 17/43 [40%] vs. *n* = 45/337 [13%], *p* < 0.001). More participants with high-order pregnancies chose to reduce or restrict their own activity levels, without formal prescription, compared to those with twin pregnancies (*n* = 33/42 [77%] vs. *n* = 206/327 [60%], *p* = 0.039).

Participants with complicated multifetal pregnancies were more frequently prescribed any type of activity restriction by a healthcare provider compared to uncomplicated pregnancies (*n* = 138/231 [60%] vs. *n* = 31/149 [21%], *p* < 0.001). Particularly, there was a greater proportion of individuals with complicated pregnancies who were prescribed bed rest compared to those with uncomplicated pregnancies (*n* = 56/138 [41%] vs. *n* = 6/31 [19%], *p* = 0.026). Additionally, there was no difference in the personal decision (without formal prescription) to reduce or restrict activity between groups (uncomplicated: *n* = 100/145 [69%] and complicated: *n* = 139/224 [62%]; *p* = 0.17).

### 3.7. Research Participation

**Figure 2:**
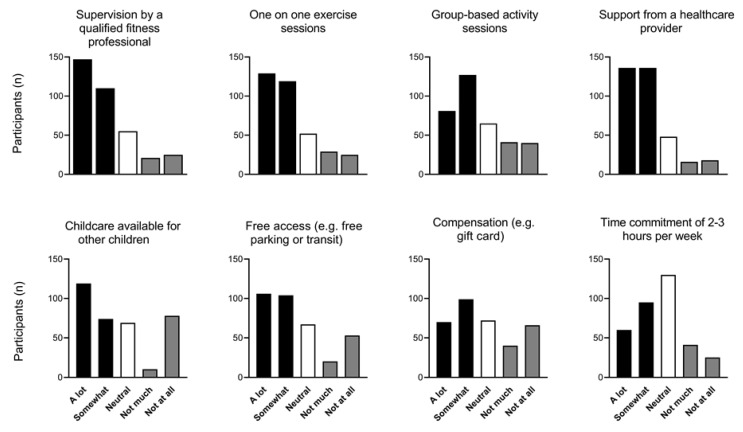
Factors that would positively influence participation in physical activity research in multifetal pregnancy.

Over half of respondents (*n* = 216/366 [59%]) reported prior participation in research (excluding this study), while only 20% (*n* = 44/216) reported participation in research during their multifetal pregnancy. If given the opportunity to participate in physical activity research during their multifetal pregnancy, 70% (*n* = 254/365) would have agreed, 17% (*n* = 61/365) responded “it depends”, and 13% (*n* = 50/365) would not have participated. Factors that would positively influence participation in physical activity research in multifetal pregnancy are shown in Figure 2. In an open-ended question about willingness to participate in research, respondents mentioned environmental/access factors (*n* = 59;, e.g., time commitment, location), accommodation/adaptation for physical symptoms (*n* = 48;, e.g., fatigue, nausea, abdominal size), psychological concerns (*n* = 29, e.g., perception of risk, body image); social (*n* = 21, e.g., support from partner/family), information (*n* = 21, e.g., what methods would be used and what activities would be involved), and need for healthcare provider approval (*n* = 14).

## 4. Discussion

The objectives of this exploratory study were to: (1) quantify physical activity behaviors in individuals with multifetal pregnancies; (2) examine barriers to physical activity in multifetal pregnancy; and (3) to generate participant-led suggestions for physical activity research in multifetal pregnancies. These data are the first to show that multifetal pregnancies do maintain physical activity while pregnant, although this may not meet current prenatal recommendations, may not be continued throughout gestation, and may not be accessible to all. The predominant barriers to prenatal physical activity in this population were physical symptoms (including fatigue/exhaustion, lower back, and pelvic pain), as well as concerns about potential risks to fetal wellbeing. Many healthcare providers prescribed activity restriction (modification or cessation of physical activity, or bed rest) to individuals with multifetal pregnancy, often without clear explanation or medical reason, and most respondents found such restrictions to be a negative experience. Most respondents would have been open to participating in prenatal physical activity research during their pregnancies, with factors such as supervision from an exercise professional and healthcare provider approval positively influencing their willingness to participate. Many respondents expressed the need for evidence-based information regarding the safety and efficacy of prenatal physical activity in multifetal pregnancy.

Multifetal pregnancies face increased risks of complications during pregnancy. Compared with singleton pregnancies, the risks of developing a hypertensive complication are doubled and preterm delivery is 12-times more likely in twin pregnancies [26]. These risks are even greater in triplet and higher-order pregnancies [27]. Such complications, as well as early hospitalization and activity restriction, pose both short and long-term burdens on the health and wellbeing of mothers, infants, and families [28]. In singleton pregnancy, physical activity is associated with multiple maternal health benefits; thus, maintaining activity in multifetal pregnancy could reduce the risks and consequences of complications in this population.

As a contraindication to prenatal physical activity in global guidelines, multifetal pregnancies have historically faced barriers to physical activity. Recently, our systematic review challenged the lack of empirical research supporting these recommendations [12]. Evidence from a small number of studies has shown that prenatal physical activity in twin pregnancy, including those with complications, may result in health benefits as well as reduced risks of adverse outcomes [12]. Specifically, twin and triplet pregnancies completing an aquatic exercise program after prescription of bed rest had improved amniotic fluid index and longer gestation in comparison to controls [29]. Additionally, twin pregnancies at risk for preterm delivery who completed a higher number of steps per day were more likely to deliver after 37 weeks compared to those with lower steps per day [30]. Even intense exercise in a rare case of a competitive marathon runner pregnant with twins had no adverse effects on either maternal or infant outcomes [31]. Furthermore, systematic management including physical activity counselling for individuals with twin pregnancies did not increase the risk of premature delivery and may reduce the risk of postpartum hemorrhage [32]. These positive previous reports, although underpowered, suggest that individuals with multifetal pregnancy can benefit from maintaining physical activity during gestation. Our data support that many individuals with multifetal pregnancies do engage with prenatal physical activity, but that significant barriers and considerations for activity prescription exist for this population.

Individuals with multifetal pregnancies reported that various physical symptoms reduced their motivation to be active during gestation; however, many of these may be improved or managed through regular movement. The most prominent symptoms limiting prenatal physical activity in multifetal pregnancy were exhaustion and fatigue. In singleton pregnancies, light to moderate resistance exercise in the second and third trimesters of pregnancy increased energy and reduced mental and physical fatigue compared to a non-exercise control group [33]. Sleep quality, which may contribute to such symptoms, has also been shown to be higher in pregnant individuals completing higher vs. lower levels of physical activity [34]. Regular physical activity also reduces the severity of lower back and pelvic girdle pain [35] and may result in a lower incidence of pregnancy-induced nausea and vomiting [36] and hyperemesis gravidarum [37]. Such results must be confirmed in those with multifetal pregnancies. However, improved education to pregnant individuals and healthcare providers regarding the benefits of physical activity on pregnancy symptom management are needed. Activities could be individually prescribed and chosen to avoid symptoms, e.g., low impact for stress incontinence or resistance exercise to avoid shortness of breath, thereby removing specific concerns and increasing comfort for those with multifetal pregnancies.

Concerns about the potential risks of prenatal physical activity were reported by over one third of respondents. To date, the safety and efficacy of physical activity in multifetal pregnancy remains understudied. However, these data provide some evidence that those with twin or high order pregnancies do engage with physical activity and want more evidence-based information regarding participation. At best, the current information regarding multifetal pregnancy and physical activity is nonspecific and an extension of findings determined in singleton gestations only. At worst, it is overprotective and based in fear. As such, encouraging and facilitating more research into physical activity during multifetal pregnancies is the only way to remove this barrier.

Accessibility or environmental barriers, such as a lack of local prenatal classes or facilities that encouraged use by individuals with multifetal pregnancies, were also prevalent. In many cases, these also tied in with concerns about body image and physical changes (e.g., abdominal size, conscious of altered/difficult movement patterns [getting on and off floor, changing clothes or putting on shoes], or difficulty using certain exercise equipment). Therefore, there may be an opportunity for online activity programs run by qualified exercise and healthcare professionals that could connect individuals with multifetal pregnancies in the comfort of their own homes. Furthermore, improving access in this way could also help to remove barriers of lack of time and/or childcare.

Severe forms of activity restriction, including avoidance of physical activity and daily activities (also known as bed rest), is commonly prophylactically prescribed for multifetal pregnancies [15]. However, this practice has no beneficial effect on preterm birth, perinatal mortality, premature rupture of membranes, or low birthweight, and can lead to psychological distress [13]. The data from this study support that many individuals with multifetal pregnancies are prescribed bed rest, as well as other forms of activity restriction, often without pre-existing conditions or diagnosis of medical complications during gestation.

Many individuals placed on bed rest during pregnancy have negative experiences, with many different emotions and stressors as a result of this restriction [38]. In this study, many participants expressed wholly negative experiences during activity restriction (including modification or cessation of physical activity, or bed rest) that had harmful impacts on maternal health and wellbeing. Specifically, many respondents described psychological impacts including prenatal depression and stress, which independently increase the odds for premature delivery [39]. Given that prenatal physical activity has been shown to reduce the odds and severity of prenatal depression [5], more work is required to critically evaluate the prescription of activity restriction/bed rest to include activities of daily living and light movement at minimum. It should be noted that some respondents found activity restriction to be positive or a combination of positive/negative; therefore, perception of this prescription was highly individualized. Healthcare providers should provide specific advice for physical activity on a case-by-case basis, but activities of daily living should be maintained [12].

In line with current global guidelines where high-order pregnancy is considered an absolute contraindication to prenatal physical activity [9,10,11], the prescription of activity restriction, particularly bed rest, was more frequent in this group compared with twin pregnancies. However, there were no differences in self-reported frequency, intensity, duration, and volume of physical activity between groups. In fact, 53% of respondents with either a triplet or quadruplet pregnancy reported being able to complete some physical activity during their gestation. These and other data suggest that some individuals with high-order pregnancies (in the absence of other contraindications) wish to be active and want support to do so. As such, high-order pregnancy should not be an absolute reason to cease activity during pregnancy; this barrier should be removed to allow for improved discussion and management of physical activity between the individual and their healthcare provider [12]. High-order pregnancies could be managed on a case-by-case basis with activity recommendations specific to those who want to and who are able to stay active. Both twin and high-order pregnancies face similar physical and psychosocial barriers to remaining active and therefore these groups could be included in research together. However, consideration of addressing specific barriers for each individual should be a key priority.

To date, no controlled investigations addressing prenatal physical activity in multifetal pregnancies have been completed. This is likely due in combination to low prevalence of multifetal pregnancies and greater concerns regarding maternal and fetal health. Before specific guidelines can be developed, empirical studies are required to ascertain the safety and efficacy of prenatal physical activity in this population. However, it is imperative that this population is consulted in the pursuit of evidence-based guidelines so that lived experiences direct research priorities and design [40]. In this study, individuals with multifetal pregnancies provided information about their interest and willingness to participate in prenatal physical activity research. The respondents demonstrated that certain factors, such as supervision by a qualified fitness professional, support from healthcare provider, childcare/free access, as well as consideration of physical symptoms would have increased the likelihood of their participation in prenatal physical activity research. Additionally, our data show the characteristics of physical activities (frequency, intensity, duration, and modality) that were achievable for individuals with both twin and high-order multifetal pregnancies. Our hope is that researchers will use this patient-derived information to aid the conception and design of future research in multifetal pregnancies.

### Considerations

This questionnaire was not validated, was self-administered, and relied upon self-reported, recall data. As the data could not be verified, there is likely reduced precision as well as bias in responses. Consequently, the data should be interpreted with this consideration. This study was an exploratory, descriptive study that was designed to encourage and facilitate physical activity research in individuals with multifetal pregnancies. Future work should include validated and objective measurements of physical activity.

The specific focus of this questionnaire was related to exercise behaviors and occupational, transportation, household or caregiving physical activities were not considered. Activities of daily living are a valid form of physical activity and are particularly important in populations where structured exercise activities are unachievable due to contraindications or logistics. Future work should seek to understand the contributions of sedentary behavior, sleep, and physical activity across the continuum in multifetal pregnancy.

Recruitment for this questionnaire was achieved through snowball and convenience sampling. As such, random sampling did not occur. However, this was an effective and feasible method of gaining respondents to this survey (*n* = 415). Most respondents were Caucasian, highly educated and delivered their multifetal pregnancy in predominantly English-speaking countries (Canada [*n* = 204/412, 50%], United States [*n* = 88, 21%], United Kingdom [*n* = 62, 15%], New Zealand [*n* = 36, 9%] and Australia [*n* = 11, 3%]). Future work should seek to include a more diverse population.

Finally, this survey was conducted during the COVID-19 pandemic. For respondents who were currently pregnant (*n* = 33/415 [8%]), government health advice may have impacted physical activity behaviors [24]. Specifically, seven participants reported reduced physical activity levels due to the closure of recreational fitness facilities; however, one participant reported higher levels of physical activity because of more time/energy and working at home.

## 5. Conclusions

Individuals with multifetal pregnancies have low engagement with current prenatal physical activity guidelines; however, many remain physically active in some capacity. There are medical, physical, and psychosocial barriers to remaining active in multifetal pregnancy and how these can be removed should be a key priority for future research. Importantly, there is a desperate need for empirical research into the safety and benefits of regular physical activity in this population. Such information could be used to support those with multifetal pregnancies who want, and are able to, stay active.

## Figures and Tables

**Table 1:** Participant demographics. Data presented as mean ± SD or *n* (%).

Maternal age at delivery (years)	32.5 ± 4.6
Gravidity	2 ± 1
Pre-pregnancy BMI (kg/m^2^)	26.2 ± 6.2
Underweight (<18.5 kg/m^2^)	7 (2%)
Normal weight (18.5–24.9 kg/m^2^)	174 (49%)
Overweight (25–29.9 kg/m^2^)	100 (28%)
Obese (>30 kg/m^2^)	75 (21%)
Gestational weight gain (kg)	16.5 ± 10.1
*Ethnicity (n = 397) †*	
Asian	12 (3%)
Black or African American	2 (1%)
Caucasian	361 (91%)
First Nations, Inuit, Métis, American Indian, or Alaska Native	9 (2%)
Hispanic of Latinx	9 (2%)
Native Hawaiian or Other Pacific Islander	2 (1%)
Mixed Heritage	9 (2%)
Prefer not to say	3 (1%)
*Gender (n = 415)*	
Female	408 (98%)
Male	5 (1%)
Nonbinary	1 (<1%)
Prefer not to say	1 (<1%)
*Education (n = 413)*	
High school	26 (6%)
College	2 (0%)
Trade, technical, or vocational training	35 (8%)
University certificate/diploma	52 (13%)
Undergraduate degree	169 (41%)
Postgraduate degree	89 (22%)
Professional degree	38 (9%)
*Pregnancy type*	
*Zygosity (n = 415)*	
Monozygotic	113 (27%)
Dizygotic	276 (67%)
Combination (high-order pregnancies only)	14 (3%)
Unsure	12 (3%)
*Chorionicity (n = 414)*	
Monochorionic	91 (22%)
Dichorionic	299 (72%)
Combination (high-order pregnancies only)	12 (3%)
Unsure	12 (3%)
*Pregnancy complications (n = 401) †*	
No complications	165 (41%)
Preterm labor	71 (18%)
Preeclampsia	60 (15%)
Gestational hypertension	43 (11%)
Gestational diabetes mellitus	45 (11%)
Short cervix (with and without cervical cerclage)	29 (7%)

† Participants could make multiple selections. Additionally, 3% of respondents were diagnosed with placenta previa, prenatal anxiety and/or cholestasis; 2% were diagnosed with placental abruption, prenatal depression, and/or subchorionic hemorrhage; ≤1% were diagnosed with hyperemesis gravidarum, anemia, premature rupture of membranes, vasa previa or peripartum cardiomyopathy.

**Table 2:** Self-reported physical activity prior to and during multifetal pregnancy.

	*n* (%)	MET Minutes Per Week Mean ± SD
*Prepregnancy physical activity (n = 406)*		
“In the year prior to pregnancy, would you describe yourself as physically active?” †		
Yes, most, if not all, of the time	174 (43%)	
Yes, sometimes	144 (35%)	
Yes, but rarely	46 (11%)	
Yes, but never meeting recommendations	24 (6%)	
No	18 (4%)	
*Prenatal physical activity (n = 388)*		
“During your multiple pregnancy, would you describe yourself as physically active?” ‡		
Yes, most, if not all, of the time	64 (16%)	
Yes, sometimes	95 (24%)	
Yes, but rarely	63 (16%)	
Yes, but never meeting recommendations	62 (16%)	
No	104 (27%)	
*Self-reported prenatal physical activity characteristics*		*n* = 300
Intensity of activity (METs)		4.5 ± 2.3
Frequency of activity (per week)		3.2 ± 1.9
Duration of activity (minutes)		43 ± 34
Volume of activity (MET·mins·week^−1^)		577 ± 758
Gestational age at cessation (weeks)		30 ± 9
*Intensity of self-reported prenatal physical activity (n = 677 responses from 300 participants)*		
Light (light effort:, e.g., yoga, easy walking, bowling, stretching)	411 (61%)	387 ± 402
Moderate (not exhausting, medium effort:, e.g., fast walking, tennis, easy bicycling, breaststroke swimming)	230 (34%)	733 ± 646
Strenuous (high effort:, e.g., running, jogging, front crawl swimming, cycling uphill)	35 (5%)	1774 ± 2125
*Location of prenatal physical activity (n = 490)*		
In a facility (e.g., gym, recreation center, yoga studio)	167 (34%)	
At home	129 (26%)	
In local neighborhood	85 (17%)	
At swimming pool	67 (14%)	
In a public park or green space	31 (6%)	
At a sporting facility (e.g., golf course, soccer field)	11 (2%)	

† Participants were given the following information alongside this question: Current guidelines recommend that all healthy adults should achieve 150-min of moderate-intensity or 75-min of strenuous-intensity physical activity per week. ‡ Participants were given the following information alongside this question: Current guidelines recommend that all healthy pregnant persons (without contraindications) should achieve 150-min of moderate-intensity physical activity per week.

**Table 3:** Barriers and physical symptoms to physical activity in multifetal pregnancy.

		*n* (%)
*Barriers (n = 363) *†		
I did not experience any barriers		49 (13%)
Pregnancy symptoms limited motivation		204 (56%)
Worried about potential risks		128 (35%)
Lack of time		86 (24%)
Healthcare provider advised against certain forms of activity		83 (23%)
Stress, anxiety, and/or low mood		45 (12%)
Weather		44 (12%)
Lack of childcare		38 (10%)
Lack of information about physical activity		35 (10%)
Lack of access to a gym or equipment		28 (8%)
Healthcare provider was unsure about physical activity		16 (4%)
Lack of support from others in life		5 (1%)
*Physical symptoms affecting physical activity levels (n = 383)* †		
No symptoms affected my physical activity levels		20 (5%)
Fatigue		274 (72%)
Exhaustion/being over-tired		219 (57%)
Lower back pain		199 (52%)
Pelvic pain		185 (48%)
Pregnancy-induced sickness (nausea, vomiting)		167 (44%)
Shortness of breath		159 (42%)
General body aches or pains (not specific to lower back or pelvis)		143 (37%)
Dizziness/light-headedness		96 (25%)
Stress incontinence		24 (6%)
Contractions		10 (3%)
Swelling of lower limbs		6 (2%)
*Overcoming barriers (n = 153)* ‡		
Theme	Example quotes	
Social	• People being more understanding of the effects of exercise on multiple pregnancy and stop assuming that multiple pregnancy will be complicated-makes it so much more stressful. • A group of moms to be who were also experiencing twin pregnancy would have been nice.	28 (18%)
Information	• More consistent advice on how much and what types of exercise were beneficial and also which would increase risks (in multifetal pregnancy). • Clearer and more conclusive information on physical activity during a multiple pregnancy.	28 (18%)
Environmental/Access	• (There was) a lack of pools that encouraged prenatal swimming or had classes. I found it difficult (in …) to find a pool that had public swimming at an early time that also encouraged preggos (pregnant individuals) to come on down. • Exercise classes specific to multiple pregnancy.	28 (18%)
Healthcare provider support	• Connection with others having the same experience, more specific information re benefits and risks, more complete advice from doctor. • More discussion with my OB (obstetrician) would have helped.	19 (12%)

† Participants could make multiple selections if they did not select “I did not experience any barriers” or “No symptoms affected my physical activity levels”. ‡ Responses could be coded to multiple themes. Other identified themes from a minority of respondents (≤5%) were psychological, economic, work, and prior experience considerations.

**Table 4:** Experience and prominent themes related to activity restriction in multifetal pregnancy.

	Top Themes	*n* (%)	Example Quotes
*Participant expressed a negative experience*		144 (61%)	
	Psychological	139 (97%)	• It was very difficult. Limited activities lead to depression and feelings of inadequacy. (I had) too much time to focus on pregnancy complications. • I knew that moving my body was a way that I regulated my mood, but I didn’t realize how much it mattered.
	Physical	96 (67%)	• When I went on medical leave, I felt physically worse (…). I just wanted the pregnancy to be over at that point. • Frustrating because I didn’t want to gain excess weight or lose muscle mass because I knew I would be physically restricted postpartum (prolapse, diastasis recti and time issues).
	Previous experience	24 (17%)	• I had been very active prior to getting pregnant. It was hard to go from essentially working out every day to not being able to even walk down my stairs. My mental health took a hit as I used exercise as a stress relief. • Struggled with the emotional and mental aspect of my body not being able to do what it previously could and the need (to) try and keep some time for me.
*Participant expressed a positive experience*		62 (26%)	
	Psychological	57 (92%)	• I emotionally felt better because I was “allowed” to take it easy. • I was fine with it. I knew it was temporary and for a good reason. It actually gave me some peace of mind.
	Physical	45 (73%)	• I was physically more at ease • It felt like my body was telling me to slow down and take care of my babies, so I rested and listened.
	Work	6 (10%)	• I was relieved. I was able to take maternity leave a few weeks earlier and not worry about being on my feet all day and possibly jeopardizing the pregnancy we had worked so hard for.
*Participant both positive and negative aspects to their experience*		32 (13%)	
	Psychological	32 (100%)	• Initially hard, but I knew what was at stake. I knew I could get my fitness back, but if anything happened to these sweet babies, I wouldn’t be able to cope. • It was hard but I remember thinking I would do anything to keep my babies safe and in as long as possible.
	Physical	27 (84%)	• Felt guilty for not exercising but knew it felt too hard on my body at the time. • I felt useless and restless, but it felt safer for my babies because I was not having so many contractions anymore.
	Social	6 (19%)	• I was able to spend time with family and friends which helped keep my mood positive. • I was okay with it but concerned about caring for my toddler.

Responses could be coded to multiple themes. Other identified themes from a minority of respondents (<15%) were environmental/access factors, sources of information, and economic considerations. Data presented in Table S5.

## Data Availability

The data presented in this study are available on request from the corresponding author.

## Supplementary Materials

The following are available online at https://www.mdpi.com/article/10.3390/ijerph18083907/s1, Table S1: Knowledge and beliefs of recommended frequency, intensity, duration, and modality of prenatal physical activity in multifetal pregnancies; Table S2: Self-reported physical activity prior to and during twin versus high-order multifetal pregnancy; Table S3: Self-reported physical activity prior to and during healthy versus complicated multifetal pregnancy; Table S4: Barriers and physical symptoms to physical activity in twin versus high-order multifetal pregnancy; Table S5: Experience and associated themes related to activity restriction in multifetal pregnancy.
