# Supplementary material for: Physical Activity Behaviors and Barriers in Multifetal Pregnancy: What to Expect When You’re Expecting More
Source: Int J Environ Res Public Health. 2021 Apr 8;18(8):3907. doi: 10.3390/ijerph18083907 (PMC8068193; doi:10.3390/ijerph18083907)
Supplement: Supplementary file 1 [file ijerph-18-03907-s001.zip › SDC - Questionnaire.docx]

Physical Activity in Multiple Pregnancy: The PA+ Questionnaire

# Information Sheet

**Why am I being asked to take part in this research study?**

You are invited to participate in this survey that aims to better understand physical activity in women who were or are pregnant with twins, triplets, or more.

**What is the reason for doing the study?**

In women pregnant with a single baby, we know that staying active during pregnancy can reduce the odds of developing complications like diabetes, high blood pressure or excessive weight gain, without any risk to the baby. Although physical activity recommendations exist for women pregnant with a single baby, we don’t know much about exercise in women who are pregnant with multiple babies. Therefore, we want to hear the experiences of women who are/were pregnant with twins, triplets, or more, so that we can better support prenatal physical activity in these groups.

**Who can take part in this study?**

Only women who are currently or were previously pregnant with multiple babies (twins, triplets, or higher) can take part in this study.

If you have been affected by self-isolation or quarantine practices in light of the COVID-19 outbreak, please answer this questionnaire thinking about your usual physical activity **before** these restrictions were put in place.

**What will happen in this study?**

If you decide to take part in the research project, you will be asked to complete an online questionnaire, which will take approximately 20 to 25 minutes. You will be asked questions about your multiple pregnancy and physical activity.

**What are the benefits to me?**

You are not expected to benefit directly from being in this research study. However, the results of this study may help other women pregnant with twins, triplets, or more in the future.

**What are the risks to me?**

There are no known risks to you participating in this study. You do not have to answer questions if you do not wish to do so.

**Do I have to take part in the study?**

Participation in this project is voluntary. It is completely up to you whether or not you participate.

**Will my information be kept private?**

During the study, you are sharing some basic information about your health with us. All of the data collected is submitted anonymously and no personal identifiable information relating to this study will be released or published by the researchers. We will do everything we can to make sure that this data is kept private.

**Will I be paid to be in the research?**

You will not be paid for participating in this study.

**What if I have questions?**

Approval for this project has been granted by a Research Ethics Board at the University of Alberta, Edmonton, Canada (Pro00093321).

Questions regarding participants’ rights should be directed to the University of Alberta Research Ethics Office at +1(780)492-2615. This office is independent of the study investigators. If you have any questions about the research now or later, please contact Dr. Margie Davenport at [margie.davenport@ualberta.ca](mailto:margie.davenport@ualberta.ca) who will respond as soon as possible.

Statement on Inclusivity:

Throughout this questionnaire, the words ‘woman/women’ may be used for convenience, as the great majority of people who have been pregnant with multiple babies self-identify as women. We encourage anyone who has been pregnant with multiple babies, regardless of their gender identification, to participate in this survey.

**Please download and retain a copy of the consent information for your records**

****PDF version of the above consent information is linked, including the six compulsory consent questions below****

# Consent

Please read through the following questions and check the box if you agree. By checking these boxes and completing the questionnaire, you imply consent to use the data provided for research purposes.

**COMPULSORY QUESTIONS:**

Do you understand that you have been asked to be in a research study?

Have you read the Information Sheet (the information provided above)?

Do you understand the benefits and risks involved in taking part in this research study?

Do you understand that you do not have to answer questions if you do not wish to do so?

Do you understand that you are free to leave the study at any time?

Do you understand that the information that you share with us will be used for research purposes only and will remain confidential?

# Questionnaire Eligibility Assessment

This section will ask a few questions to ensure your eligibility to complete this survey.

**Questions 1&2 are compulsory :**

1. Are you currently pregnant?
   1. Yes, with a multiple pregnancy (twins, triplets or more)
   2. Yes, with a single baby pregnancy
      1. If this answer is selected, branched question: Have you had a multiple baby pregnancy in the past?
         1. Yes
         2. No
   3. No, I have had a multiple baby pregnancy in the past
   4. No, I have only ever had a single baby pregnancy in the past
   5. No, I have never been pregnant before
2. If answer yes to currently pregnant: How many weeks pregnant are you right now with your multiple baby pregnancy?

Questions 3& 4 are non compulsory

1. How many overall pregnancies (not babies) have you had? A current pregnancy counts as one pregnancy.
   1. #__
2. Have you had more than one multiple pregnancy (twins, triplets, or more)?
   1. Yes
      1. If select yes, the following message pops up:

For the purposes of the current survey, please answer using information from only the most recent of your multiple baby pregnancies. However, we would like to gather data for all of your multiple pregnancies. If you are willing, once you have submitted this survey, please re-take the survey and answer with regards to your other multiple baby pregnancy.

- 1. No

Question 5 is compulsory

1. How many babies were you pregnant with during your multiple pregnancy?
   1. Twins
   2. Triplets
   3. Quadruplets
   4. Quintuplets
   5. Sexuplets
   6. Septuplets or higher

**ALL QUESTIONS FOLLOWING ARE VOLUNTARY:**

1. Were your babies:
   1. Identical (Monozygotic) OR Fraternal (Dizygotic) OR Combination OR not sure
   2. Monochorionic (sharing a placenta) OR Dichorionic/Polychorionic (NOT sharing a placenta) OR Combination OR not sure
2. If your babies are a combination of zygosity and/or chorionicity, please describe. Please use lettered labels for each baby in your description (e.g. Baby A and Baby B)

# Maternal Characteristics

In this section, we would like to know some general information about you and your health, specifically during your multiple pregnancy.

1. With which gender identity do you most identify?
   1. Female
   2. Male
   3. Not listed/Other, please explain: ____
   4. Prefer not to say
2. What is your highest level of education completed:
   1. None
   2. Elementary school
   3. Some High school, no diploma
   4. High school
   5. Trade/technical/vocational training
   6. University certificate/diploma
   7. University Bachelor’s degree
   8. University Masters’ degree
   9. University Doctoral degree
   10. Professional Degree (MD, DMD, DDS, Law, etc).
   11. Other, please specify:___
   12. None
   13. Prefer not to say
3. In what country were you born?
   1. Country drop down list
4. In what country did you/will you deliver your babies?
   1. Country drop down list
5. What is your ethnicity? (Select all that apply)
   1. Asian
   2. Black or African American
   3. Caucasian
   4. Hispanic or Latinx
   5. First Nations, Mètis, Inuit, American Indian, or Alaska Native
   6. Native Hawaiian or other Pacific Islander
   7. Mixed Heritage
   8. Unknown
   9. Other, please explain: ____
   10. Prefer not to say
6. How old were you when you delivered your [multiple_type]? Please answer in years
   - 1. The coding of the square brackets will insert the respondent’s answer from question 3
   1. ___ years
7. How old are you now? Please answer in years.
   1. ___Years
8. How did you conceive your [multiple_type]?
   1. Spontaneous (without the use of reproductive technology)
   2. With help from assistive reproductive technology (ART)

Note: Assistive reproductive technology includes methods such as hormonal assistance, IVF, and others.

1. The next two questions are regarding weight change during your multiple pregnancy. Please choose a unit of measure to answer in:
   1. Pounds
   2. Kilograms
   3. I don’t remember how much I weighed before and during my multiple pregnancy
2. How much did you weigh immediately prior to becoming pregnant?
   1. #__kg/lbs
3. How much did you weigh at the end of pregnancy? If currently pregnant, how much do you weigh right now?
   1. #___kg/lbs
4. Please ensure that the following is correct:
   1. I weighed [prepreg_weight] kg/lbs before I was pregnant.
   2. I weighed [prepreg_weight_3] kg/lbs at the end of my pregnancy (or right now if I am pregnant).
      1. This coding will insert the respondent’s answers from above with the correct unit of measure for the participant to verify
5. The next question is about your height, would you rather answer in centimetres (cm) or feet and inches?
6. How tall are you in centimetres/ feet and inches?
   1. #___
7. Please ensure that the following is correct:
   1. I am [matchar_height_cm] cm/ft + in tall.
      1. This coding will insert the respondent’s answers from above with the correct unit of measure for the participant to verify
8. Were you diagnosed with any of the following complications during or after your multiple pregnancy? Please only answer on behalf of your health, we will ask about your babies’ outcomes later.
   1. Gestational Diabetes Mellitus No Yes, weeks: _____
   2. Impaired Glucose Tolerance/Pre-diabetes No Yes, weeks: _____
   3. Gestational Hypertension /high blood pressure No Yes, weeks: _____
   4. Preeclampsia No Yes, weeks: _____
   5. Eclampsia No Yes, weeks: _____
   6. Placenta Previa No Yes, weeks: _____
   7. Preterm Labour No Yes, weeks: _____
   8. Short cervix No Yes, weeks: _____
   9. Cervical Cerclage (or stitch) No Yes, weeks: _____
   10. Placental abruption No Yes, Weeks:_____
   11. Prenatal depression No Yes, weeks: _____
   12. Prenatal Anxiety No Yes, Weeks:_____
   13. Postnatal depression No Yes, weeks: _____
   14. Postnatal anxiety No Yes, Weeks:_____
   15. Other, Which other condition(s) were you diagnosed with during your multiple pregnancy?
       Please list the week you were diagnosed at: _______________. Separate additional conditions with a semi-colo (;).
   16. I was not diagnosed with any complications
9. Were you diagnosed with any other medical conditions before your multiple pregnancy?
   1. No, I had no pre-existing medical conditions
   2. Anxiety
   3. Autoimmune disease (excluding MS)
   4. Bipolar disorder
   5. Cancer
   6. Cardiovascular condition
   7. Chronic high blood pressure
   8. Depression
   9. Epilepsy
   10. Multiple Sclerosis
   11. Categorized as Overweight or Obese
   12. Respiratory disease
   13. Type 1 diabetes
   14. Type 2 diabetes
   15. Other condition(s), please explain ________

# Infant Characteristics

In this section, we would like to know some information about your babies and your delivery.

1. How many weeks pregnant were you when you gave birth to your [multiple_type]?
   1. ___
2. How did you deliver your babies (select all that apply)?
   1. Spontaneous delivery
   2. Planned delivery
   3. Emergency delivery
   4. Not yet delivered
3. Were any of your babies admitted to the neonatal intensive care unit (NICU) at birth?
   1. Yes
   2. No
4. If yes: How many of your babies were admitted to the NICU? ____
5. Did any of your babies experience any of the following complications during or following pregnancy:
6. My babies were not diagnosed with any complications
7. Breathing problems
8. Congenital disorder
9. Small for gestational age (<10^th^ percentile)
10. Large for gestational age (>90^th^ percentile)
11. Infection
12. Jaundice
13. Neonatal hypoglycaemia
14. Restricted growth
15. Twin-to-twin transfusion syndrome
16. Other baby complications, please explain: ____

# Physical Activity Knowledge

In this section, we would like to know about what you knew about prenatal physical activity when you were pregnant with [multiple_type]. We would only like to know what you thought was appropriate to do during an uncomplicated multiple pregnancy, whether or not this is what you actually did.

1. Thinking back to when you were pregnant with [multiple_type]: In general, how often did you think that you should be physically active each week? Please answer what you thought, whether or not you were able to do this.
2. One time per week
3. Two times per week
4. Three times per week
5. Four times per week
6. Five times per week
7. Six times per week
8. Every day of the week
9. Other time per week, please explain: ____________
10. Thinking back to when you were pregnant with [multiple_type]: In general, what intensity/which intensities did you think you should exercise at? Please answer what you thought, whether or not you were able to do this. Select all that apply.
11. I did not think I should be physically active during my multiple baby pregnancy
12. Light Intensity (light effort; e.g. yoga, easy walking, bowling, stretching).
13. Moderate Intensity (not exhausting, medium effort; e.g. fast walking, tennis, easy bicycling, breast stroke swimming)
14. Strenuous Intensity (high effort; e.g. running, jogging, front crawl swimming, cycling uphill).
15. Other intensity, please explain: ____________
16. Based on the intensity/intensities you selected above: In general, how many minute per week (total) did you think you should be active for at this intensity/these intensities? Please answer what you thought, whether or not you were able to do this. If you did not think you should be active, write 0.
17. ___ minutes per week
18. Thinking back to when you were pregnant with [multiple_type]: In general, what type of activities did you think were okay to do during you pregnancy? Please answer what you thought, whether or not you did these activities. Select all that apply.
19. Walking
20. Jogging
21. Running
22. Indoor cycling
23. Outdoor cycling
24. Dance
25. Snowshoeing
26. Cross country skiing
27. Downhill skiing
28. Swimming
29. Other water based activities
30. Body weight exercise
31. Resistance exercise using machines
32. Resistance exercise using free weights
33. Yoga
34. Pilates
35. Stretching
36. Other, please explain: ____
37. I did not think any of these activities were okay to do
38. I did not think any activity was okay to do

# Physical Activity Advice and Sources

In this section we would like to know what kind of information you knew about physical activity during your multiple baby pregnancy.

1. Did your healthcare provider talk with you about prenatal exercise and/or physical activity during your multiple baby pregnancy?
2. Yes, my healthcare provider openly discussed physical activity with me
3. Yes, but I had to ask my healthcare provider for information about exercise/physical activity
4. No, I did not talk about physical activity during pregnancy with my healthcare provider Other: ____________
5. I cannot remember
6. If select yes, which healthcare provider(s) talked with you about being physically active during your multiple pregnancy? Select all that apply
7. Family doctor
8. Obstetrician
9. Nurse
10. Midwife
11. Other healthcare provider, please explain _________
12. If select yes or other, what advice were you given by your doctor or other healthcare provider(s)?
13. Short answer
14. Did you, or were you able to, follow this advice? Please explain.
15. Short answer
16. Did you use any other resources to find information on physical activity wile pregnant? Select all that apply
17. Books
18. Social media
19. Family or friends
20. In person pregnancy class or support group
21. Exercise professional (e.g. personal trainer)
22. Exercise governing bodies (e.g. CSEP, ACSM, SMA, etc.)
23. Obstetrician governing bodies (e.g. BJOG, ACOG, JOGC, etc.)
24. Healthcare websites (e.g. NHS, NIH, WebMD, Mayo Clinic, etc.)
25. Other sites or search engines on the internet
26. Other, please explain: ___
27. I did not use any other resources to look for information on exercise and pregnancy
28. What, if any, advice did you find?
29. Short answer
30. Were you able to find information about physical activity that was specific to having a multiple pregnancy? Please explain.
31. Short answer

## Pre-pregnancy physical activity

In this section, we would like to know about how active you were PRIOR to your multiple pregnancy?

1. **In the year prior to** your pregnancy would you describe yourself as physically active? (Current recommendations are at least 150 minutes per week of moderate-intensity or 75-minutes of strenuous-intensity physical activity per week.)
   1. Yes, most, if not all, of the time meeting recommendations
   2. Yes, sometimes meeting recommendations
   3. Yes, but rarely meeting recommendations
   4. Yes, but never reaching recommendations
   5. No
   6. Other, please explain: __________

# Prenatal physical activity

*In this section, we would like to know about how active you were DURING your multiple pregnancy.*

1. **During** **your multiple pregnancy,** would you describe yourself as physically active? (Current recommend that all healthy pregnant persons (without contraindications) should achieve 150-minutes of moderate-intensity physical activity per week.)
2. Yes, most, if not all, of the time meeting recommendations
3. Yes, sometimes meeting recommendations
4. Yes, but rarely meeting recommendations
5. Yes, but never reaching recommendations
6. No
7. Other, please explain: __________

If yes:

The next series of questions are about the kinds of physical activities you may have done during your multiple pregnancy.

You will be able to add up to 5 separate kinds of activities.

Please consider all activities you did **regularly**, even if you had to stop them part way through your pregnancy.

Use the following definitions to aid in describing your physical activity types:

Light Intensity (light effort; e.g. yoga, easy walking, golf, bowling, stretching).

Moderate Intensity (not exhausting, medium effort; e.g. fast walking, baseball, tennis, easy bicycling, breast stroke swimming)

Strenuous Intensity (high effort; e.g. running, jogging, fast front crawl swimming, cycling uphill).

1. How many activities would you like to add (0 to 5 maximum) (questions a-f below will appear 0-5 times dependent on the participant’s selection)
2. Type of physical activity (short answer)
3. How many times per week did you do this activity?
4. On average, how long did you spend doing this activity (in minutes)?
5. Based on the definitions given earlier, what was the intensity of this activity?
   - 1. Light
     2. Moderate
     3. Strenuous
6. Where did you complete this activity? (short answer)
7. At any point in your pregnancy did you have to stop or reduce your participation in this activity? If yes, when? Why?
   - 1. Short answer
8. What were some of your favourite exercise types to do while pregnant? Please explain why.
9. _____
10. What were some of your least favourite exercise types to do while pregnant? Please explain why.
11. Short answer ____
12. Whether you were active or not, did any of the following symptoms stop you from being active/more active during your pregnancy? Select all that apply.
13. Fatigue
14. Pelvic pain
15. Lower back pain
16. Stress incontinence
17. Shortness of breath
18. Exhaustion/being over-tired
19. Dizziness/light-headedness
20. Pregnancy-induced sickness (nausea, vomiting)
21. General body aches or pains (not specific to your lower back)
22. Other, please explain: _____
23. No symptoms affected my physical activity levels
24. Is there anything else you would like to add about your physical activity habits or experiences during your pregnancy?
25. Open ended

# Barriers to Physical Activity

In this section, we would like to know if there were any reasons that prevented you from being physically active during your multiple baby pregnancy.

1. Were there any barriers to you being physically active during your multiple pregnancy? Select all that apply:
2. I did not experience any barriers
3. Lack of time
4. Poor weather
5. Lack of child care
6. Stress, anxiety and/or low mood
7. I was worried about potential risks
8. Lack of access to a gym or equipment
9. Lack of support from others in your life
10. Lack of information about physical activity
11. Pregnancy symptoms limited my motivation
12. My healthcare provider advised me against certain forms of activity
13. My healthcare providers was unsure about me being physically active
14. Other, please explain: ___
15. If answer yes to any barriers: Is there anything that would have helped you to overcome these barriers?
16. Short answer
17. Was there anything you wish that you had known about physical activity during your multiple pregnancy:
18. Short answer

# Activity Restrictions

In this section, we would like to know about whether you were told to restrict your activity levels during your multiple pregnancy.

1. During your multiple pregnancy, were you ever prescribed to restrict your activity levels by a healthcare provider? Restricting activity would mean avoiding specific activities like exercise, walking, and/or sexual activity. This may have been called bed rest.
2. Yes
3. No
4. What kind of activity restriction were you prescribed? Select all that apply.
5. Restrict your physical activity intensity, duration, or frequency
6. Avoid physical activity
7. Be on pelvic rest (e.g. avoidance of lower body exercise or sexual activity)
8. Avoid all activity apart from walking to the washroom
9. Other activity restriction, please explain: Please include what week of your multiple pregnancy you were told to do this.
   - 1. Short answer___
10. All activity restriction answers above branch to a questions about the week of pregnancy this recommendation was given at
11. Do you know why you were prescribed this activity restriction?
12. Yes, please explain why: ____
13. No, I don’t know
14. No, I don’t remember
15. Other, please explain: ____
16. During your [multiple_type] pregnancy, did you ever reduce your activity by choice?
17. Yes, please explain why: ___
18. No
19. Don’t remember
20. Other, please explain: ____
21. How did you find the experience of activity restriction? Describe your physical, emotional, and/or mental experience of being prescribed activity restriction.
22. Open ended

# Other Factors Influencing PA

In this section we will ask a few more questions related to your experiences and attitudes towards physical activity during your multiple pregnancy.

## Prior Pregnancies:

1. Since having your multiple pregnancy, have your opinion about physical activity during pregnancy changed?
2. Yes
3. No
4. Other
5. Please explain:
6. Open ended
7. If you have had other pregnancies, were there differences in your lifestyle, anxieties, or physical activity habits from your multiple pregnancy?
8. Open ended

# Pregnancy Research Participation

This section will ask you about any prior research participation and gather your insights on pregnancy and exercise research, specifically with regards to pregnancies with multiple babies.

1. Have you ever participated in any research study? *Not including this questionnaire
2. Yes
3. No
4. If yes: did you participate in any research study while you were pregnant with multiple babies?
5. Yes
6. No
7. If you had the opportunity to participate in a physical activity research study during your multiple pregnancy would you have said yes?
8. No
9. Yes
10. It depends/other
11. If C: What would your participation depend on?
12. Open ended
13. We would like to know what factors might encourage participation in physical activity research by those pregnant with multiple babies.
    - 1. Please rate the factors below as to how much they would have impacted your ability, or interest, in being part of a physical activity research during your multiple pregnancy: (ranking is: not at all, not much, neutral, somewhat, a lot)
14. Supervision by a qualified fitness professional
15. One on one exercise
16. Group-based activity sessions
17. Support from a healthcare provider
18. Childcare available for other children
19. Free access (e.g. free parking or transit reimbursement)
20. Small compensation for participation (e.g. gift card)
21. Time commitment of 2-3 hours per week
22. Are there any other factors that would have influenced your ability, or willingness, to participate in a research study during your multiple pregnancy?
23. Open ended
24. Do you have any reasons for ***not*** participating in a research study during your multiple pregnancy?
25. Open ended
26. Is there anything else you would like us to know?
27. Open ended

**Thank you for completing this questionnaire.**
