# Supplementary material for: Physical Activity Behaviors and Barriers in Multifetal Pregnancy: What to Expect When You’re Expecting More
Source: Int J Environ Res Public Health. 2021 Apr 8;18(8):3907. doi: 10.3390/ijerph18083907 (PMC8068193; doi:10.3390/ijerph18083907)
Supplement: Supplementary file 1 [file ijerph-18-03907-s001.zip › SDC - Table 1.docx]

**Supplemental Digital Content – Table 1.** Knowledge and beliefs of recommended frequency, intensity, duration, and modality of prenatal physical activity in multifetal pregnancies.

|  | | **n (%)** |
| --- | --- | --- |
| *Prenatal physical activity frequency* (n=414) | |  |
| “During your multiple pregnancy, how often did you think that you should be physically active each week?” | | |
|  | Every day of the week | 116 (28%) |
|  | 6 times per week | 6 (1%) |
|  | 5 times per week | 55 (13%) |
|  | 4 times per week | 53 (13%) |
|  | 3 times per week | 98 (24%) |
|  | 2 times per week | 29 (7%) |
|  | 1 time per week | 13 (3%) |
|  | I did not think I should be active | 34 (8%) |
|  | Other | 10 (2%) |
| *Prenatal physical activity intensity* (n=412) † | |  |
| “During your multiple pregnancy, what intensity/which intensities did you think you should exercise at?” | | |
|  | Light (light effort: e.g., yoga, easy walking, bowling, stretching) | 270 (66%) |
|  | Moderate (not exhausting, medium effort: e.g., fast walking, tennis, easy bicycling, breaststroke swimming) | 148 (36%) |
|  | Strenuous (high effort: e.g., running, jogging, front crawl swimming, cycling uphill) | 12 (3%) |
|  | I did not think I should be active at any intensity | 28 (7%) |
| *Prenatal physical activity duration* (n=404) | |  |
| “In general, how many minutes per week (total) did you think you should be active?” | | |
| Duration (minutes) *Presented as mean±SD* | | 164±206 |
|  | I did not think I should be active | 26 (6%) |
|  | Under 150-minutes | 198 (49%) |
|  | 150-minutes or more | 180 (45%) |
| *Prenatal physical activity modalities* (n=413) † | |  |
| “Select all activities that you thought were ok to do during your multiple pregnancy.” | | |
|  | Walking | 402 (97%) |
|  | Swimming | 307 (74%) |
|  | Stretching | 305 (74%) |
|  | Yoga | 286 (69%) |
|  | Pilates | 167 (40%) |
|  | Other water-based activities | 161 (39%) |
|  | Body weight exercise | 153 (37%) |
|  | Indoor cycling | 141 (34%) |
|  | Dance | 139 (34%) |
|  | Jogging | 119 (29%) |
|  | Resistance exercise using free weights | 112 (27%) |
|  | Resistance exercise using machines | 102 (25%) |
|  | Outdoor cycling | 88 (21%) |
|  | Snowshoeing | 64 (15%) |
|  | Cross country skiing | 53 (13%) |
|  | Running | 45 (11%) |
|  | Other (including suggestions of golf, rowing, elliptical and team sports) | 14 (3%) |
|  | Downhill skiing | 4 (1%) |
|  | I did not think any of these activities were okay to do | 4 (1%) |
|  | I did not think any activity was okay to do | 4 (1%) |

† Participants could make multiple selections.
