# Supplementary material for: Physical Activity Behaviors and Barriers in Multifetal Pregnancy: What to Expect When You’re Expecting More
Source: Int J Environ Res Public Health. 2021 Apr 8;18(8):3907. doi: 10.3390/ijerph18083907 (PMC8068193; doi:10.3390/ijerph18083907)
Supplement: Supplementary file 1 [file ijerph-18-03907-s001.zip › SDC - Table 2.docx]

**Supplemental Digital Content – Table 2.** Self-reported physical activity prior to and during twin *versus* high-order multifetal pregnancy.

|  | | **Twin pregnancy** | **High-order pregnancy** | ***P* value** |
| --- | --- | --- | --- | --- |
| *Pre-pregnancy physical activity* | |  |  |  |
| “In the year prior to pregnancy, would you describe yourself as physically active?” † | | *n*=360 | *n* =47 |  |
|  | Yes, most, if not all, of the time | 157 (44%) | 17 (36%) | 0.33 |
|  | Yes, sometimes | 131 (36%) | 13 (28%) | 0.24 |
|  | Yes, but rarely | 39 (11%) | 7 (15%) | 0.41 |
|  | Yes, but never meeting recommendations | 19 (5%) | 5 (11%) | 0.14 |
|  | No | 14 (4%) | 4 (9%) | 0.15 |
| *Prenatal physical activity* | |  |  |  |
| “During your multiple pregnancy, would you describe yourself as physically active?” ‡ | | *n*=345 | *n* =43 |  |
|  | Yes, most, if not all, of the time | 60 (17%) | 4 (9%) | 0.18 |
|  | Yes, sometimes | 88 (26%) | 7 (16%) | 0.18 |
|  | Yes, but rarely | 56 (16%) | 7 (16%) | 0.99 |
|  | Yes, but never meeting recommendations | 57 (17%) | 5 (12%) | 0.41 |
|  | No | 84 (24%) | 20 (47%) | 0.002 |
|  |  |  |  |  |
| Self-reported prenatal physical activity characteristics § | | *n*=278 | *n* =22 |  |
|  | Intensity of activity (METs) § | 4.3±1.4 | 4.0±1.2 | 0.43 |
|  | Frequency of activity (per week) § | 4.0±1.5 | 3.3±1.4 | 0.17 |
|  | Duration of activity (minutes) § | 42±26 | 32±13 | 0.17 |
|  | Volume of activity (MET·mins·week^-1^) § | 1312±1521 | 1174±1052 | 0.73 |
|  | Gestational age at cessation (weeks) § | 30±8 | 27±10 | 0.17 |
|  | Achieving over 500 MET·mins·week^-1^ | 196 (71%) | 12 (55%) | 0.12 |

Statistical comparisons made using test of two proportions.

† Participants were given the following information alongside this question: *Current guidelines recommend that all healthy adults should achieve 150-minutes of moderate-intensity or 75-minutes of strenuous-intensity physical activity per week.*

‡ Participants were given the following information alongside this question: *Current guidelines recommend that all healthy pregnant persons (without contraindications) should achieve 150-minutes of moderate-intensity physical activity per week.*

§ Statistical comparisons made using multiple t-tests. The false discovery rate was determined using the Two-stage linear step-up procedure of Benjamini, Krieger and Yekutieli, with Q = 5%. Each parameter was analyzed individually, without assuming a consistent SD.
