# Supplementary material for: Physical Activity Behaviors and Barriers in Multifetal Pregnancy: What to Expect When You’re Expecting More
Source: Int J Environ Res Public Health. 2021 Apr 8;18(8):3907. doi: 10.3390/ijerph18083907 (PMC8068193; doi:10.3390/ijerph18083907)
Supplement: Supplementary file 1 [file ijerph-18-03907-s001.zip › SDC - Table 3.docx]

**Supplemental Digital Content – Table 3.** Self-reported physical activity prior to and during healthy *versus* complicated multifetal pregnancy.

|  | | **Uncomplicated pregnancy** | **Complicated pregnancy** | ***P* value** |
| --- | --- | --- | --- | --- |
| *Pre-pregnancy physical activity* | |  |  |  |
| “In the year prior to pregnancy, would you describe yourself as physically active?” † | | *n*=164 | *n* =243 |  |
|  | Yes, most, if not all, of the time | 79 (48%) | 95 (39%) | 0.69 |
|  | Yes, sometimes | 55 (34%) | 89 (37%) | 0.52 |
|  | Yes, but rarely | 13 (8%) | 33 (14%) | 0.08 |
|  | Yes, but never meeting recommendations | 9 (5%) | 15 (6%) | 0.77 |
|  | No | 8 (5%) | 10 (4%) | 0.71 |
| *Prenatal physical activity* | |  |  |  |
| “During your multiple pregnancy, would you describe yourself as physically active?” ‡ | | *n*=154 | *n* =234 |  |
|  | Yes, most, if not all, of the time | 36 (23%) | 28 (12%) | **0.003** |
|  | Yes, sometimes | 44 (29%) | 51 (22%) | 0.13 |
|  | Yes, but rarely | 23 (15%) | 40 (17%) | 0.56 |
|  | Yes, but never meeting recommendations | 22 (14%) | 40 (17%) | 0.46 |
|  | No | 29 (19%) | 75 (32%) | **<0.001** |
|  |  |  |  |  |
| Self-reported prenatal physical activity characteristics | | *n*=124 | *n* =175 |  |
|  | Intensity of activity (METs) § | 4.4±1.4 | 4.2±1.4 | 0.11 |
|  | Frequency of activity (per week) § | 3.3±1.4 | 3.4±1.5 | 0.24 |
|  | Duration of activity (minutes) § | 45±30 | 38±20 | **0.013** |
|  | Volume of activity (MET·mins·week^-1^) § | 1625±1957 | 1073±987 | **0.003** |
|  | Gestational age at cessation (weeks) § | 31±8 | 29±8 | **0.016** |
|  | Achieving over 500 MET·mins·week^-1^ | 96 (77%) | 113 (65%) | **0.017** |

Statistical comparisons made using test of two proportions. † Participants were given the following information alongside this question: *Current guidelines recommend that all healthy adults should achieve 150-minutes of moderate-intensity or 75-minutes of strenuous-intensity physical activity per week.*

‡ Participants were given the following information alongside this question: *Current guidelines recommend that all healthy pregnant persons (without contraindications) should achieve 150-minutes of moderate-intensity physical activity per week.*

§ Statistical comparisons made using multiple t-tests. The false discovery rate was determined using the Two-stage linear step-up procedure of Benjamini, Krieger and Yekutieli, with Q = 5%. Each parameter was analyzed individually, without assuming a consistent SD.
