# Supplementary material for: Physical Activity Behaviors and Barriers in Multifetal Pregnancy: What to Expect When You’re Expecting More
Source: Int J Environ Res Public Health. 2021 Apr 8;18(8):3907. doi: 10.3390/ijerph18083907 (PMC8068193; doi:10.3390/ijerph18083907)
Supplement: Supplementary file 1 [file ijerph-18-03907-s001.zip › SDC - Table 4.docx]

**Supplemental Digital Content – Table 4.** Barriers and physical symptoms to physical activity in twin *versus* high-order multifetal pregnancy.

|  | Twin pregnancy | High-order pregnancy | *P-*value |
| --- | --- | --- | --- |
| *Barriers* † | *n*=321 | *n*=42 |  |
| I did not experience any barriers | 42 (13%) | 7 (17%) | 0.52 |
| Pregnancy symptoms limited motivation | 185 (58%) | 19 (45%) | 0.13 |
| Worried about potential risks | 109 (34%) | 19 (45%) | 0.15 |
| Lack of time | 82 (26%) | 4 (10%) | **0.021** |
| Healthcare provider advised against certain forms of activity | 64 (20%) | 19 (45%) | **<0.001** |
| Stress, anxiety, and/or low mood | 42 (13%) | 3 (7%) | 0.27 |
| Weather | 43 (13%) | 1 (2%) | **0.039** |
| Lack of childcare | 34 (11%) | 4 (10%) | 0.83 |
| Lack of information about physical activity | 30 (9%) | 5 (12%) | 0.60 |
| Lack of access to a gym or equipment | 26 (8%) | 2 (5%) | 0.45 |
| Healthcare provider was unsure about physical activity | 13 (4%) | 3 (7%) | 0.36 |
| Lack of support from others in life | 4 (1%) | 1 (2%) | 0.55 |
| *Physical symptoms affecting physical activity levels* † | *n*=341 | *n*=42 |  |
| No symptoms affected my physical activity levels | 19 (6%) | 1 (2%) | 0.38 |
| Fatigue | 245 (72%) | 29 (69%) | 0.70 |
| Exhaustion/being over-tired | 192 (56%) | 27 (64%) | **0.006** |
| Lower back pain | 177 (52%) | 22 (52%) | 0.95 |
| Pelvic pain | 164 (48%) | 21 (50%) | 0.81 |
| Pregnancy-induced sickness (nausea, vomiting) | 147 (43%) | 20 (48%) | 0.58 |
| Shortness of breath | 146 (43%) | 13 (31%) | 0.14 |
| General body aches or pains (not specific to lower back or pelvis) | 125 (37%) | 18 (43%) | 0.44 |
| Dizziness/light-headedness | 88 (26%) | 8 (19%) | 0.34 |
| Stress incontinence | 20 (6%) | 4 (10%) | 0.36 |
| Contractions | 8 (2%) | 2 (5%) | 0.35 |
| Swelling of lower limbs | 6 (2%) | 0 (0%) | 0.38 |

† Participants could make multiple selections if they did not select “I did not experience any barriers” or “No symptoms affected my physical activity levels.” Statistical comparisons made using test of two proportions.
