# Supplementary material for: Physical Activity Behaviors and Barriers in Multifetal Pregnancy: What to Expect When You’re Expecting More
Source: Int J Environ Res Public Health. 2021 Apr 8;18(8):3907. doi: 10.3390/ijerph18083907 (PMC8068193; doi:10.3390/ijerph18083907)
Supplement: Supplementary file 1 [file ijerph-18-03907-s001.zip › SDC - Table 5.docx]

**Supplemental Digital Content - Table 5.** Experience and associated themes related to activity restriction in multifetal pregnancy.

| Experience of activity restriction | Top themes | n (%) | Example quotes |
| --- | --- | --- | --- |
| *Participant expressed a negative experience* | | 144 (61%) |  |
|  | Social | 22 (15%) | - Most challenging was when the restrictions impacted my interactions with my toddler. That was very upsetting. |
|  | Work | 17 (12%) | - I felt frustrated. I didn't think the physical activity restriction was necessary once I was no longer working, but also didn't want to risk preterm labor so I felt a little stuck. I was bored. |
|  | Environmental/Access | 13 (9%) | - Because [when] the belly grew, it was difficult to fit it between my thighs and my torso on the bike. Because it was winter, so walking fast on the ice might have been more dangerous. |
|  | *No references to economic or information sources were identified.* | | |
| *Participant expressed a positive experience* | | 62 (26%) |  |
|  | Previous experience | 4 (6%) | - Stopped playing sports like netball, soccer etc. for safety. Happy to do so for the sake of babies. |
|  | Environmental/Access | 1 (2%) | - Hot weather (100-110 [in …] in July/ August) |
|  | Information | 1 (2%) | - I decided together with my personal trainer to stop any activity that would make me out of breath. Because I read that if I'm out of breath, it would impact the oxygenation for babies. |
|  | *No references to social or economic themes were identified.* | | |
| *Participant both positive and negative aspects to their experience* | | 32 (13%) |  |
|  | Work | 4 (113%) | - Physically grateful, I didn't realize how much strain simple things like going to work was having on my body until I stopped doing it. Mentally it was hard because I didn't want to stop being activity. |
|  | Previous experience | 2 (6%) | - I was depressed and just frustrated that I couldn't do any physical activity [but] I had multiple losses in my past and just wanted to carry these babies to term. |
|  | Environmental/Access | 1 (3%) | - When I was unable to continue to do yoga in a gym setting, I switched to water yoga which boosted my mood. |
|  | Economic | 1 (3%) | - I was disappointed that I was unable to utilize my yoga gym membership through to the end of my pregnancy |
|  | *No references to information sources were identified.* | | |
